# Shaping Lycopene Nanoparticles Performance: How Surfactants Influence Stability, Antioxidant Activity, and Uptake in Human Skin Spheroids

**DOI:** 10.3390/antiox15010136

**Published:** 2026-01-21

**Authors:** Francesca Baldassarre, Chiara Boncristiani, Michela Ottolini, Concetta Nobile, Maryam Shahzad Shirazi, Giuseppe E. De Benedetto, Gianpiero Colangelo, Viviana Vergaro, Ludovico Valli, Giuseppe Ciccarella

**Affiliations:** 1 Department of Biological and Environmental Sciences and Technologies (DiSTeBA), University of Salento, S. P. 6 Lecce-Monteroni SNC, 73100 Lecce, Italy; michela.ottolini@unisalento.it (M.O.); maryam.shahzadshirazi@unisalento.it (M.S.S.); ludovico.valli@unisalento.it (L.V.); giuseppe.ciccarella@unisalento.it (G.C.); 2 UdR INSTM, Salento, 73100 Lecce, Italy; 3 Department of Experimental Medicine, University of Salento, S. P. 6 Lecce-Monteroni SNC, 73100 Lecce, Italy; chiara.boncristiani@unisalento.it; 4 Institute of Nanotechnology, CNR NANOTEC, Consiglio Nazionale delle Ricerche, S. P. 6 Lecce-Monteroni SNC, 73100 Lecce, Italy; concetta.nobile@nanotec.cnr.it; 5 Department of Cultural Heritage, University of Salento, Via D. Birago 64, 73100 Lecce, Italy; giuseppe.debenedetto@unisalento.it; 6 Department of Engineering for Innovation, University of Salento, S. P. 6 Lecce-Monteroni SNC, 73100 Lecce, Italy; gianpiero.colangelo@unisalento.it

**Keywords:** lycopene, PLGA nanoparticles, non-ionic surfactants, human skin spheroids

## Abstract

There is a growing demand for plant-derived antioxidants to replace synthetic ones in skincare applications. Phytochemicals are characterized by certain limitations, including poor bioavailability and chemical instability, which affect their industrial exploitation. Tomato peel extract has been used as a source of lycopene, which is renowned for its antioxidant properties. To improve the bioavailability of extracted lycopene, polymeric (poly-lactic-co-glycolic acid) nano-carriers were synthesized by comparing two non-ionic surfactants, polyvinyl alcohol (PVA) and Tween 20. The impact of surfactants has been studied by evaluating: (i) colloidal stability determined by dynamic light scattering; (ii) lycopene retention and bioactivity over time, as measured by spectrophotometric assays; (iii) biological interactions on 2D and 3D keratinocyte and melanocyte cell cultures. It was found that both surfactants enable the formation of stable lycopene-loaded nanoparticles suspensions; however, greater colloidal stability was exhibited by nanoparticles prepared with Tween 20. PVA, on the other hand, provided greater nanoparticle stability in terms of loaded lycopene retention and antioxidant activity. Tween 20 surfactant improves the internalization of lycopene-loaded nanoparticles in human skin spheroids. It was demonstrated that both surfactants provided excellent intracellular antioxidant activity of lycopene. This was observed in keratinocytes, melanocytes, adherent cells, and spheroids, suggesting potential skincare applications.

## 1. Introduction

Significant advancements have been made over the past few decades in enhancing the bioavailability of bioactive ingredients for the food, cosmetics, and pharmaceutical industries [1,2]. Lycopene (Lyc) is one of the most widely commercialized carotenoids thanks to its high antioxidant capacity among natural, plant-derived active compounds (e.g., vitamins, anthocyanins, and phenolic compounds) [3,4]. In addition, among various carotenoids, Lyc is the second most potent antioxidant after astaxanthin. It is a significant inhibitor of reactive oxygen species (ROS); it has been demonstrated to remove singlet oxygen at a rate that is two and ten times higher than that of beta-carotene and alpha-tocopherol, respectively [5]. Lycopene is also recognized for its potential anti-inflammatory properties, and antidiabetic, antithrombotic, and antimicrobial effects, as well as for its protective effects on the cardiovascular system, its ability to mitigate oxidative damage, and prevent cancer [6,7]. Recent epidemiological studies have provided insights into the health benefits of dietary Lyc, which has been shown to decrease the risk and complications associated with several chronic diseases, including cardiovascular diseases, obesity, type 2 diabetes, cancer, and neurodegenerative disorders. The etiology of these chronic diseases is principally associated with oxidative stress-induced systemic and low-grade chronic inflammation. Lyc has been demonstrated to attenuate elevated levels of proinflammatory mediators (e.g., proinflammatory cytokines and oxidized phospholipids) by modulating oxidative stress, a property that is attributable to its potent antioxidant characteristics. The available evidence suggests that this combination has the capacity to act as a safeguard against the development of chronic inflammatory disorders [8]. As demonstrated in approximately 200 articles and reviews, Lyc has been shown to have anti-obesity and anti-diabetic properties in a variety of organs and tissues, including but not limited to adipose tissue and the liver, kidneys, pancreas, brain, ovaries, intestines, and eyes. The underlying mechanism of these effects can be attributed to the substance’s antioxidant and anti-inflammatory properties, as well as its ability to regulate specific signalling pathways [9]. Concerning cancer prevention, it has been demonstrated that a higher dietary intake and circulating concentration of Lyc have protective effects against prostate cancer, in a dose-dependent way [10]. Lycopene has also shown an inhibitory effect on the proliferation and progression of colorectal cancer cells [5]. Lycopene’s nutraceutical impact involves benefits for cardiovascular and neurodegenerative diseases; it can improve vascular and endothelial function, reduce atherosclerotic plaque size, and contribute to cognitive longevity and the treatment of several neuronal diseases (Parkinson’s and Alzheimer’s diseases) [11,12].

Lycopene cannot be produced by the human body and thus must be obtained through dietary intake. It is the pigment responsible for the red colouring of foods such as tomatoes, watermelons, papayas, pink grapefruits, and guavas.

Although Lyc and carotenoids can be obtained through chemical synthesis, these processes usually involve complicated, low-yield reactions that are not economically or environmentally favourable. Other strategies include extraction from plant sources and, more recently, microbial synthesis. Extraction from plants, especially tomatoes, is a conventional method that is widely performed; however, it tends to result in low yields, requires large areas of land for cultivation, and suffers from seasonality. Using microorganisms for carotenoid production is appealing because of their rapid growth, high yields, and year-round availability. However, the cost of growth media undermines the viability of this method. In this context, producing Lyc using waste products offers a dual benefit: generating sustainable, high-value products, while providing an alternative waste management solution [13,14]. In the interest of conserving resources and reducing the considerable expenses associated with waste disposal, there has emerged a novel trend of utilizing waste recovery processing residues as prospective sources of compounds with active properties, such as nutraceuticals and functional ingredients [15].

Tomatoes represent the most significant source of Lyc. Italy, along with Spain and Portugal, is one of Europe’s biggest producers of tomatoes. Every year, more than 10 million tons of these fruits are turned into peeled tomatoes, juices, and other products. This process creates a lot of waste (millions of tons each year and about 4% of the weight of the processed tomatoes). This is both an inconvenience for the producers and a resource. As Lyc is predominantly present in the skin of the fruit, tomato peel (TP) has the potential to serve as an alternative, cost-effective source of carotenoids [16]. Lycopene derived from tomato waste is generally considered safe and is gaining regulatory interest for use in food and supplements. Bodies such as the European Food Safety Authority (EFSA) have established an acceptable daily intake (ADI) of 0.5 mg/kg body weight per day. Extracts from tomato by-products are considered novel foods (EFSA’s Novel Foods Regulation), requiring specific safety assessments, especially regarding other carotenoids present. The EFSA scrutinizes new products also for the presence of phytoene and phytofluene (precursor carotenoids of Lyc) and ensures that overall intake does not exceed the ADI [17]. Specific bodies are responsible for regulating and certifying natural, organic, and naturally derived ingredients for use in cosmetics and cosmeceuticals (such as CosMOS and NATRUE). These bodies’ ultimate goal is to promote sustainable cosmetics throughout their entire production cycle, from sourcing raw materials to packaging and communication (https://www.cosmos-standard.org; https://natrue.org/it/, accessed on 7 January 2026). They also promote sustainable circular economy practices to add value to waste products. However, the safety of Lyc depends on the extraction method used.

Methods of Lyc extraction include organic solvent extraction (conventional, such as liquid–liquid, Soxhlet, microwave, and ultrasound-assisted extraction), and green techniques (alternative). In the food, cosmetic, and pharmaceutical industries, an environmentally friendly carotenoid extraction process with minimal loss of bioactivity is desirable. Supercritical CO_2_ (SC-CO_2_) extraction is a more environmentally friendly alternative to organic solvent extraction and is already used in the food industry to produce solvent-free extracts [3,18].

However, due to the high degree of unsaturation exhibited by Lyc, a highly significant propensity for chemical degradation and cis–trans isomerization is exhibited under conditions of extreme temperature and pH value, exposure to oxygen, and to light. The observed phenomenon of colour fading in the compound is primarily attributable to chemical instability. This, in turn, results in a concomitant loss of both bioavailability and bioactivity [19,20]. The exploitation of Lyc biological properties is also impeded by its hydrophobic nature and insolubility in aqueous solvents, as well as in many other solvents, which affect its efficient delivery. These limitations considerably restrict its commercial applications [21,22]. Additionally, the high degree of lipophilicity could impact its permeation through the stratum corneum (the outermost layer of the epidermis) in dermatological applications. This phenomenon can be attributed to the substance’s propensity to interact with the components of stratum corneum, resulting in its prolonged retention within this initial layer [23,24,25].

To improve the delivery of Lyc to the deeper layers of the skin, several formulation strategies have been suggested, including the use of surfactants, permeation enhancers, cyclodextrin complexes, and various nanoscale delivery systems [19,20,22]. Delivery systems such as nanoemulsions, liposomes, niosomes, nanogels, and polymeric and inorganic nanoparticles improve the stability, permeability, and targeted delivery of bioactive compounds and plant extracts [26,27,28,29]. The utilization of biopolymeric and lipid-based nanostructures as effective carriers has been demonstrated to be a promising approach for the protection of Lycopene throughout the digestive system, with the potential for incorporation into functional foods or nutraceutical products [19,20]. To enhance the delivery of Lyc to the deeper layers of the skin, vesicular nanocarriers such as liposomes and more deformable variants, including transfersomes, ethosomes and niosomes, and nanoemulsions have been proposed [24,25,30,31].

It has been established that Lyc is more efficient than β-carotene in preventing oxidative damage when applied in skincare formulations. This has been demonstrated to provide beneficial results in terms of protection against photodamage and indicates its potential as an anti-ageing ingredient [32,33,34]. Lycopene-rich products, including tomato extract obtained by SC-CO_2_, have demonstrated protective effects against UVB/UVA irradiation, and anti-inflammatory and anti-apoptosis responses on different cell lines (e.g., fibroblasts and keratinocytes) [31,35]. Previous studies have also reported anti-tyrosinase and anti-melanogenesis properties, which support the great potential of these natural extracts in dermatology [35]. In detail, the protective effects of Lyc-rich products have been demonstrated through the inhibition of NF-κB [33] and UVB-induced ornithine decarboxylase (ODC) activity, along with the prevention of inflammatory responses [34]. As was indicated in previous in vitro studies, tomato extracts have also been reported to greatly inhibit tyrosinase (73%) [36]. Furthermore, carotenoids have been demonstrated to offer protection against melanogenic intermediates and exogenous DNA damage [37], and a protective effect on the skin by preventing the apoptosis of fibroblast cells [38]. Specifically, various anti-ageing treatments, facial moisturizers, and eye creams now contain Lyc due to their great ability to protect the skin from potential UV radiation damage and prolong a tan [39,40].

In this study, Lyc, starting from TP extract in the oil form and obtained with SC-CO_2_ (provided by Licofarma s.r.l., Galatina, Lecce, Italy), has been nonencapsulated using poly-lactic-co-glycolic acid (PLGA), a copolymer that has been approved by the U.S. Food and Drug Administration, synthesizing Lyc-loaded nanoparticles for delivery in skin cells. The suitability of PLGA for bioactive encapsulation, as flavonoids and carotenoids, has been widely investigated [41,42]. Recent studies have demonstrated enhanced stability and protection against the chemical degradation of Lyc, even when subjected to simulated in vitro gastrointestinal conditions, through the utilization of nanoencapsulation by PLGA [43,44].

The objective of the study was to optimize the physicochemical and biological properties of Lyc-loaded PLGA nanoparticles (Lyc@PLGA NPs) by manipulating their stabilization phase using two distinct surfactants, namely polyvinyl alcohol (PVA) and Tween 20. Surfactants have been shown to play a pivotal role in the stabilization process during the synthesis of polymeric NPs. The impact of surfactants on the synthesis process is such that they have the capacity to affect a number of key factors, including the size of the particles, the zeta potential, the surface morphology, the drug loading and, ultimately, the biological interactions [45,46,47,48,49].

SC-CO_2_ TP extract has been compared to a Lyc standard, analyzing UV and HPLC spectra. The produced nanoparticles were characterized by electron microscopy, spectrophotometry, FT-IR, and dynamic light scattering to study their morphology, Lyc retention, and colloidal stability over time. The antioxidant capacity of TP extract and Lyc@PLGA NPs have been assessed with DPPH inhibition testing. Biological interactions have been studied in a spontaneously immortalized human keratinocyte cell line, HaCaT, and melanoma cell line, SK-MEL-2. Two-dimensional and three-dimensional cell cultures have been treated to verify the surfactant impact on cell viability, uptake, and intracellular antioxidant activity. A cellular uptake study was performed to provide direct visualization and quantification of NP internalization in human skin spheroids. This has been achieved through the utilization of confocal imaging, which involves the analysis of the intrinsic fluorescence of Lyc, thereby circumventing the necessity for additional NP chemical modifications. This approach has been applied as preliminary screening to in vitro permeation (e.g., Franz diffusion cells) and ex vivo assays on animal or human skin sections, thereby addressing methodological and ethical concerns. It has been demonstrated that 3D cell culture systems, including skin cell models, have the capacity to overcome the limitations of 2D cultures. This is due to their ability to replicate the complex phenotypical heterogeneity that occurs during cell growth [50].

Stable nanosuspensions have been produced, providing better colloidal stability with Tween 20 and higher Lyc stability over time with PVA. Cellular assays demonstrated that the internalization of Lyc@PLGA NPs in HaCaT and SK-MEL-2 spheroids was particularly affected by surfactant, rather than the antioxidant activity that is high in any case, thus contributing to the potential Lyc exploitation in skincare applications.

## 2. Materials and Methods

### 2.1. Materials

Tomato peel extract in oil form, obtained with SC-CO_2_, has been kindly provided by the cosmetics farmer Licofarma s.r.l. (Galatina, Lecce, Italy). Lycopene ≥ 98%(HPLC) from tomato, PLGA, RESOMER RG 501, polyvinyl alcohol (PVA) Mw 31,000–50,000, 98–99% hydrolyzed, Tween 20, 2,2-Diphenyl-1-picrylhydrazyl (DPPH), 2′,7′-dichlorofluorescein diacetate (DCFDA, ≥97% HPLC, powder, cell-permeable non-fluorescent probe), and thiazolyl blue tetrazolium bromide (MTT) were purchased from Merck Life Science S.r.l. (Milan, Italy). DAPI was purchased from Thermo Scientific (Rockford, IL, USA). Dulbecco’s Modified Eagle Medium (DMEM) (4.5 mg/L glucose) was purchased from Biowest (Nuaillé, France).

### 2.2. High-Performance Liquid Chromatography Analysis of Lyc in the TP Extract

For high-performance liquid chromatography (HPLC) analysis, an Agilent 1100 HPLC system (Santa Clara, CA, USA) consisting of a solvent tray, a vacuum degasser G1322A, a binary pump G1312A, an autosampler G1329A, a thermostatted column compartment G1316, and a diode array UV-vis detector G1315B was used. The system was controlled by Chemstation software (version number A.08.01 [783], Agilent).

Chromatographic separations were performed in isocratic mode at 30 °C using a Thermo Scientific Accucore C18 reversed phase column (2.6 μm, 10 cm × 2.1 mm). The detection wavelength was 472 nm. The mobile phase consisted of methanol and acetonitrile (50/50 *v*/*v*) at a flow rate of 0.3 mL/min.

### 2.3. Preparation of Lyc-Loaded PLGA Nanoparticles

Lyc@PLGA NPs were prepared modifying the emulsification solvent diffusion method described in the literature [51,52]. Briefly, 100 mg of PLGA (RESOMER RG 501, Merck Life Science S.r.l., Milan, Italy) was dissolved in 8 mL of ethyl acetate and solubilized for 2 h under agitation. Then, 12.5 mg of TP extract (measured density of 0.8 g/mL) was dissolved in 2 mL of PLGA solution to obtain a polymer (mg)/extract (mg) ratio of 2/1. This organic phase was mixed with 0.5 mL of filtered deionized water (0.45 µm filter) at 3.000 rpm for 30 min at RT. Afterwards, this first water-in-oil (*w*/*o*) emulsion was poured drop by drop using a syringe with a thin needle, into 10 mL of a 0.5% (*w*/*v*) surfactant solution, which was homogenized at 8000 rpm for 3 min, using an Ultra-Turrax blender model IKA T25 (IKA Werke, Staufen, Germany) in an ice bath. Two emulsifiers were used to compare different colloid stabilization actions, in detail: PVA (Mw 31,000–50,000, 98–99% hydrolyzed) obtaining Lyc@PLGA@PVA NPs and Tween 20 obtaining Lyc@PLGA@Tween 20 NPs. The resulting water-in-oil-in-water (*w*/*o*/*w*) emulsions were stirred overnight on a magnetic stirring plate at 500 rpm to remove the organic solvent through evaporation. The produced nanoparticles were centrifuged at 15,000 rpm for 15 min at 4 °C and washed (with deionized water) three times to remove emulsifiers. The suspension was finally suspended in filtered deionized water, freeze-dried, and stored at 4 °C for the subsequent characterizations and biological studies.

### 2.4. Characterization of Lyc-Loaded PLGA Nanoparticles

#### 2.4.1. Scanning Electron Microscopy

Morphological characterization of PLGA NPs has been performed by scanning electron microscopy, under a FESEM (MERLIN, Carl Zeiss GmbH, Oberkochen, Germany) operating at an accelerating voltage of 20 kV. A sample solution drop (10 μL) was placed on a standard TEM carbon-coated Cu grid and dried at room temperature.

#### 2.4.2. Dynamic Light Scattering Analysis

Lyc@PLGA NPs’ colloidal stability and size distribution were investigated by average hydrodynamic diameter, polydispersity index, and ζ-potential measurements through dynamic light scattering (DLS). Samples were analyzed with a Nano ZS90 (Malvern Instruments, Malvern, UK) instrument. The ζ-potential analysis was carried out by laser doppler velocimetry (LDV). Measurements were performed at 25 °C, in filtered distilled water (0.45 μm), at a concentration of 0.05 mg/mL, setting the refractive index of PLGA (1.33). The ζ-potential values are reported as the mean of 5 measurements; each of them was derived from 20 different runs to establish measurement repeatability. Particle size distribution data are reported as the mean of 3 measurements (each of them derived from 12 different runs) to warrant repeatability. Lyc@PLGA@PVA NPs and Lyc@PLGA@Tween 20 NPs were analyzed after synthesis and after 25 days of storage in aqueous solution at 4 °C.

#### 2.4.3. UV–Vis Assay

Lycopene content in TP extract and PLGA NPs was determined by UV-vis spectrophotometry, scanning from 350 to 550 nm by a Jasco V-530 spectrophotometer (JASCO EUROPE s.r.l., Cremella, Italy). The samples were prepared in ethyl acetate. TP extract has been dissolved in the solvent at the concentration of 0.3 mg/mL. The Lyc loaded into PLGA NPs was extracted under NP dissolution by vortexing 2 mg of each sample in 1 mL of ethyl acetate for 3 min and sonicating in an ice bath for 30 min. The resulting solutions were then centrifuged at 15,000 rpm for 10 min at 4 °C before UV-vis analysis. The quantification has been performed referring to a standard curve using a Lyc standard from tomato (Merck Life Science S.r.l.) at known concentrations in the range 1.5–25 µg/mL in ethyl acetate; measurements of Abs values at λ_max_ 485 nm were chosen, and the line fitting has been performed through Origin software (version 93E) [31,53].

#### 2.4.4. Fourier-Transform Infrared Spectroscopy

The vibrational features of the samples were analyzed by FT-IR spectroscopy. The direct analysis on Lyc@PLGA@PVA NPs and Lyc@PLGA@Tween 20 NPs was performed comparing PLGA (as a blank control) and Lyc@PLGA NPs in their freeze-dried form, whereas the TP extract was measured as a solution in ethyl acetate. A small aliquot of each sample was deposited onto the ATR element of an FT-IR spectrometer (PerkinElmer Spectrum One); the TP extract was allowed to evaporate completely prior to acquisition, while the freeze-dried nanoparticle powders were analyzed directly. The analysis on Lyc released by PLGA NPs was performed following NP dissolution as described in the previous Section 2.4.3. For each measurement, 64 scans were collected in the spectral range 4000–600 cm^−1^. All spectra were recorded under identical instrumental conditions to enable qualitative comparison.

### 2.5. Determination of Free Radical Scavenging Activity

Free radical scavenging activity has been determined by quantifying the inhibition of the DPPH reagent. TP extract was dissolved in ethyl acetate at a concentration of 6 mg/mL and then diluted in the working solutions to analyze DPPH inhibition to achieve the selected quantities of 0.25–0.5–1–2 mg. The scavenging activity of Lyc loaded into PLGA NPs was determined following NP dissolution as described in the previous Section 2.4.3. Briefly, 0.5 mL of the produced samples (TP extract and Lyc solution released by PLGA NPs) was added to 2.5 mL of 0.1 mM DPPH-methanolic solution and vigorously shaken in the dark at room temperature. The absorbance of samples at 515 nm was measured after 30 min. Ascorbic acid and methanol were used as positive and negative controls, respectively. Ascorbic acid (2 mg in 0.5 mL of water) was added to 2.5 mL of a 0.1 mM methanolic DPPH solution, in the same way as for the extract. The scavenging activity was calculated using Equation (1):

(1) $$SA\%=\;\left[\frac{Abs\;control-Abs\;sample}{Abs\;control}\right]\times \;100$$

where *control* is the DPPH-methanolic solution.

### 2.6. Stability of Lyc-Loaded PLGA NPs

For the storage stability study, the powders of Lyc@PLGA@PVA NPs and Lyc@PLGA@Tween 20 NPs were kept at 4 °C for 5 months and analyzed for any change in Lyc content and percent residual scavenging activity [54]. Lyc content has been quantified at each time interval (0–2–3–5 months) using the procedure just described in Section 2.4.3. The retention index percentage has been calculated using the following Equation (2):

(2) $$Retention\;index\;(\%)=\frac{C1}{C0}\;\times 100$$

where C1 represents the Lyc content after storage (2–3–5 months), and C0 is the Lyc content before storage, at time 0 (after freeze-drying).

The retentional antioxidant was calculated using the following Equation (3):

(3) $$Scavenging\;activity\;retention\;rate\;(\%)=\frac{D1}{D0}\;\times 100$$

where D1 is the DPPH radical scavenging activity of the sample at different intervals of storage (2–3–5 months), and D0 is the DPPH radical scavenging activity of the sample before storage, at time 0 (after freeze-drying). Sample preparation and scavenging activity were performed as described in Section 2.5.

### 2.7. In Vitro Biological Studies of Lyc-Loaded PLGA NPs

#### 2.7.1. Cell Culture and Spheroids Formation

HaCaT (obtained from CLS Cell Lines Service GmbH (Eppelheim, Baden-Wurttemberg, Germany) and SK-MEL-2 cell lines (ATCC (Manassas, VA, USA; HTB-72)) were maintained in Dulbecco’s modified Eagle’s medium (DMEM) supplemented with fetal bovine serum (FBS) (10%), penicillin (100 U·mL^−1^ culture medium), streptomycin (100 mg·mL^−1^ culture medium), and glutamine (5%). Cells were grown in a humidified incubator at 37 °C, 5% CO_2_, and 95% relative humidity.

For the spheroid formation, HaCaT and SK-MEL-2 were trypsinized and seeded into each well of an ultra-low attachment U-shaped 96-well plate (Corning Costar 7007, Merck Life Science S.r.l., Milan, Italy) in 80 μL complete medium at cell densities of 12 × 10^4^ cells/well and 8 × 10^4^ cells/well, respectively. The U-plates were centrifuged at 3000 rpm for 20 min at 4 °C, and then the plates were transferred in the humidified incubator for at least 24 h to allow the spheroid formation and growth. The morphological characteristics of the spheroids, including their diameter and shape, were determined by microscopic optical analysis using an inverted EVOS XL Cell Imaging System microscope (Thermo Fisher, Waltham, MA, USA).

#### 2.7.2. Cell Viability Test by MTT Assay

Cells were added to 24-well culture plates at 1 mL per well, and incubated at 37 °C, 5% CO_2_, and 95% relative humidity for 24, 48, and 72 h with Lyc@PLGA@PVA NPs and Lyc@PLGA@Tween 20 NPs (50–100–200 µg/mL). The control was a complete culture medium. After the incubation period, cultures were removed from the incubator, and MTT solution (5 mg/mL) was aseptically added. Cultures were returned to the incubator and incubated for 3 h. Afterwards, the cultures were removed from the incubator, and the resulting MTT formazan crystals were dissolved in DMSO. The plates were ready within 15 min after adding DMSO. After the incubation time, pipetting up and down was required to completely dissolve the MTT formazan crystals. Absorbance at a wavelength of 570 nm was measured using a Microplate Reader CLARIOstar PLUS (BMG LABTECH, Ortenberg, Germany). Calculate the percentage of cell viability using Equation (4):

(4) $$Cells\;Viability\;(\%)=\;\frac{Abs\;(t)}{Abs\;(nt)}\;\times \;100$$

where Abs (t) is the absorbance of treated cells and Abs (nt) is the absorbance of non-treated cells. The results are expressed as mean ± SD of three separate experiments.

Spheroid viability upon incubation with Lyc@PLGA@PVA NPs and Lyc@PLGA@Tween 20 NPs was measured by MTT assay, as described by Bresciani et al., 2019 [55]. Briefly, on the third day, spheroids covered by 80 μL of medium were incubated with NP suspensions at different concentrations (50, 100, and 200 µg/mL). After 24 h of incubation, the medium was removed, the spheroids were washed with PBS, and 100 μL of 5 mg/mL MTT solution was added to each well. After 3 h of incubation, the MTT solution was removed, the insoluble formazan crystals were dissolved with 100 μL of dimethyl sulfoxide, and the absorbance at 570 nm was measured in a Microplate Reader CLARIOstar PLUS. Cell viability percentage was calculated using Equation (4). The results are expressed as mean ± SD of three separate experiments, in which four spheroids were used for each condition.

#### 2.7.3. Cellular Uptake of Lyc-Loaded PLGA Nanoparticles by Confocal Imaging

HaCaT (12 × 10^4^ cells) and SK-MEL-2 (8 × 10^4^ cells) spheroids were seeded as explained above and treated with nanoparticle suspension with a concentration of 100 µg/mL. After rinsing with phosphate-buffered saline (PBS) twice, spheroids were fixed with PFA 4% for 20 min at RT, followed by three washes with PBS, then stained with DAPI for 1 h. The stained spheroids were stored in PBS at 4 °C until imaging. The spheroids were imaged as z stacks with a Zeiss LSM700 confocal microscope (Zeiss, Oberkochen, Germany) equipped with a Zeiss Axio Observer Z1 inverted microscope using a 10× and 20× objective with a 1.46 numerical aperture for imaging using the same settings between repeats. Laser beams with 405 nm and 488 nm excitation wavelengths were used for DAPI and FITC for lycopene imaging, respectively. Spheroid volume was calculated using ImageJ v1.51n software with the 3D Object Counter v2.0 plugin. The threshold for background and object voxels was manually adjusted for each image in order to capture the whole volume of each spheroid [56,57].

#### 2.7.4. Intracellular Reactive Oxygen Species Measurement by DCFDA Assay

A total of 3 × 10^5^ HaCaT cells and 2 × 10^5^ SK-MEL-2 cells were seeded into 6-well culture plates with 2 mL of media per well and incubated for 72 h in order to reach a final confluence of 80%. Both cell lines were first treated for 24 h at 37 °C with the PLGA NP suspensions and, simultaneously, with H_2_O_2_ to induce exogenous stress (1 mM). Twenty-four hours later, the cells were exposed to a 10 µM DCFDA solution for 30 min. After that, period cells were collected, washed twice with PBS, and subjected to centrifugation at 1200× *g* for 5 min at room temperature. Subsequently, the cell pellet was resuspended in PBS supplemented with 1% FBS and the cells were analyzed. The results are expressed as mean ± SD of three separate experiments.

Three-day-old 3D spheroids of HaCaT (12 × 10^4^ cells/well) and SK-MEL-2 (8 × 10^4^ cells/well) were selected for reactive oxygen species (ROS) analysis. These spheroids were exposed for 24 h at 37 °C to PLGA NP suspensions supplemented with 1 mM H_2_O_2_. After the incubation period, all spheroids were rinsed with 10 mL of PBS and were collected in a 15 mL tube. After centrifugation (5 min, 400× *g*), spheroids were incubated for 30 min with DCFDA solution (10 µM), washed twice with PBS, and subjected to centrifugation at 1200× *g* for 5 min at room temperature. Subsequently, the cell pellet was resuspended in PBS supplemented with 1% FBS and the cells were analyzed. The results are expressed as mean ± SD of three separate experiments, in which 12 spheroids were used for each condition.

Flow cytometry was performed on a flow cytometer (BD Biosciences, San Jose, CA, USA) equipped with Cytexpert software (version 2.6).

## 3. Results

### 3.1. Characterization of TP Extract

**Figure 1:**
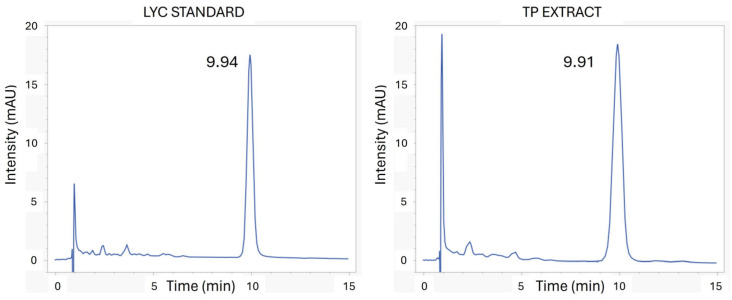
HPLC chromatograms (λ = 472 nm) of Lyc standard and TP extract in ethyl acetate.

TP extract (Licofarma s.r.l., Galatina, Italy) and Lyc standard, both in ethyl acetate, have been analyzed by HPLC. As illustrated in Figure 1, the chromatogram of TP extract shows a peak at retention time (Rt) of 9.91 min, in line with the peak at Rt 9.94 min of the Lyc standard.

**Figure 2:**
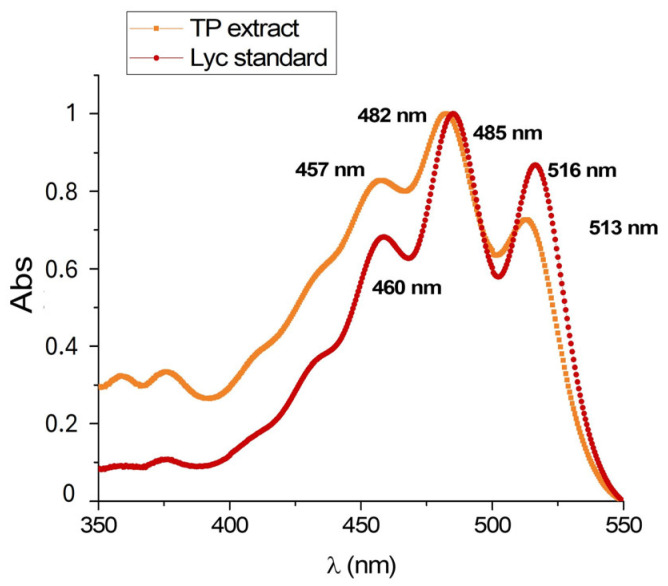
UV-vis spectrum of TP extract and Lyc standard in ethyl acetate.

This comparison has also been made with UV-vis. As we can observe in Figure 2, the absorption spectrum of Lyc has been recorded in the extract solubilized in the same solvent.

Lycopene from TP extract obatained with SC-CO_2_ (Licofarma s.r.l.) showed three absorbance peaks (λ_max_) at 457, 482, and 513 nm, consistent with those obtained for the Lyc standard from tomato (Merck Life Science S.r.l.) (460 nm, 485 nm, and 516 nm).

Bioactivity in terms of free radical scavenging activity has been determined by DPPH inhibition test, testing TP extract solubilized in ethyl acetate. As demonstrated in Figure S1, the antioxidant activity increases linearly with an increasing amount of dissolved extract. At 2 mg, a percentage of 96 is achieved, which is equivalent to the activity observed for the same amount of ascorbic acid, which was utilized as a positive control.

### 3.2. Charaterization of Lyc@PLGA NPs

#### 3.2.1. Influence of Surfactants on NP Physicochemical Properties

DLS analysis has been used to study the surfactant influence on particle size, zeta potential, and colloidal stability of Lyc@PLGA NP aqueous suspensions. Table 1 reports the ζ-potential, average hydrodynamic diameter (Z-average), and polydispersity index (PdI) of Lyc@PLGA@PVA NPs and Lyc@PLGA@Tween 20 NPdiluted suspensions.

The ζ-potential values ≥ |20 mV| and PDI ≤ 0.5 indicated stable colloidal suspensions for both the used surfactant. The utilization of Tween 20 produced particles with a more negative charge and a lower average hydrodynamic size compared to PVA. The improved size distribution due to Tween 20 is evident in the intensity plot shown in Figure S2. No difference was measured between the PDIs, indicating that both suspensions have good homogeneity. A higher negative charge is also found in particles produced with Tween 20 when empty particles are produced under the same conditions without the addition of the extract, as shown in Table S1. However, the PLGA@Tween 20 NPs’ |ζ-potential| is lower than the |ζ-potential| of Lyc@PLGA@Tween 20 NPs (≅−30 vs. ≅−44 mV). No differences in terms of average size and polydispersity have been registered for empty NPs. Furthermore, the colloidal stability is maintained for 25 days in an aqueous suspension, as indicated by the unaltered DLS parameters reported in Table S2.

**Figure 3:**
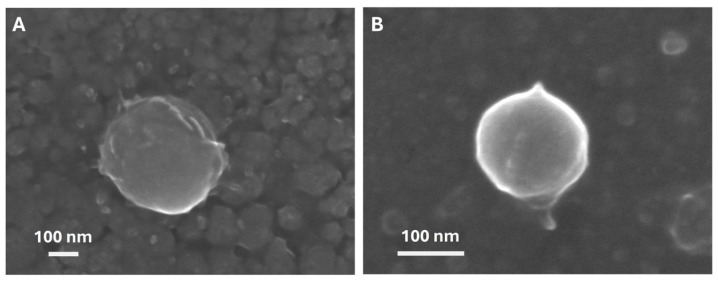
Representative SEM images at different magnifications of a single particle, for (**A**) Lyc@PLGA@PVA NPs and (**B**) Lyc@PLGA@Tween 20 NPs.

Morphological analysis by SEM confirmed the formation of spherical particles with real dimensions in the range 100–200 nm, as can be observed in representative SEM images in Figure 3 and Figure S3.

#### 3.2.2. Influence of Surfactants on Lyc Encapsulation and Stability

**Figure 4:**
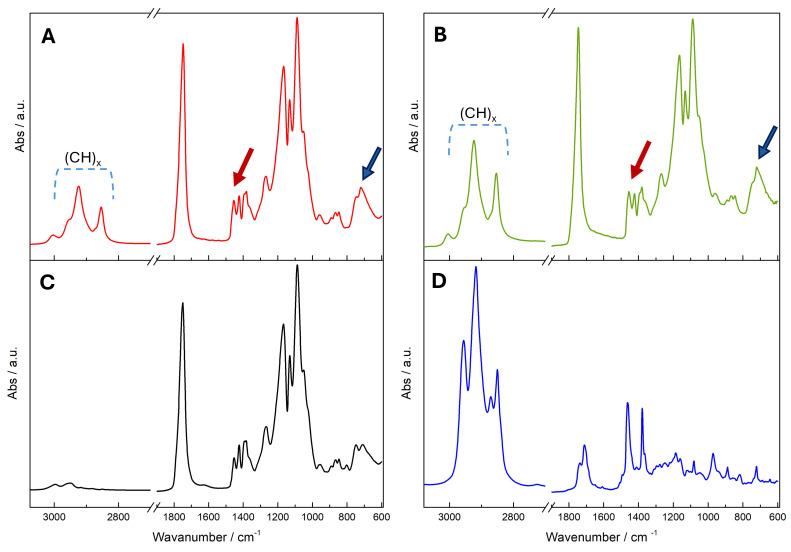
FT-IR spectra of Lyc@PLGA@PVA-NPs (**A**), Lyc@PLGA@Tween20-NPs (**B**), PLGA (**C**) and TP extract in ethyl acetate (**D**). Dashed rectangles indicate the aliphatic C–H stretching region (3000–2800 cm⁻¹). Red arrows denote the conjugated C=C stretching vibrations of lycopene (1600–1500 cm⁻¹), while blue arrows highlight the characteristic out-of-plane C–H bending modes of the lycopene polyene chain in the 850–750 cm⁻¹ region.

The qualitative verification of Lyc encapsulation was obtained through FT-IR analysis conducted on Lyc@PLGA NPs, blank PLGA, and the TP extract. In Figure 4, organized into four single-spectrum panels (Figure 4A–D), the vibrational profiles are presented separately in order to clearly highlight the spectral regions associated with Lyc. Panels A and B show the spectra of freeze-dried NPs, Lyc@PLGA@PVA NPs (red line), and Lyc@PLGA@Tween 20 NPs (green line), respectively, whereas panels C and D report the FT-IR spectra of PLGA (black line) and the TP extract analyzed in ethyl acetate (blue line). In panels A and B, the blue dashed rectangles mark the aliphatic C–H stretching region (3000–2800 cm^−1^) associated with (C–H) groups typical of carotenoids and clearly visible in the TP extract (panel Figure 4D). The red arrows indicate the conjugated C=C stretching vibrations (1600–1500 cm^−1^), which represent the fundamental signature of the polyene chain of Lyc. The blue arrows highlight the region between 850 and 750 cm^−1^, where Lyc exhibits characteristic out-of-plane C–H bending and torsional vibrations of the polyene backbone—features that are not observed in blank PLGA. These Lyc-associated bands appear in both nanoparticle formulations but are absent in the blank PLGA (panel Figure 4C), which display only the typical polymer-related vibrations, including the strong ester C=O stretching band at 1750 cm^−1^ and the C–O–C stretching region between 1300 and 1100 cm^−1^.

The integrity of Lyc after release from the nanoparticles was further evaluated by analyzing the spectra of the formulations dissolved in ethyl acetate, as shown in Figure S4. The spectra of the dissolved NPs exhibited the full set of Lyc-associated features, including the aliphatic C–H stretching (3000–2800 cm^−1^), the conjugated C=C stretching modes (1600–1500 cm^−1^) and, notably, the characteristic band at 960 cm^−1^, attributed to the out-of-plane =C–H bending of all-trans Lyc (see Figure S4).

FT-IR spectra features have also been verified by UV-vis analysis. In Figure S5, UV-vis spectra of Lyc solution following NP dissolution at time 0 are reported. By comparing these spectra with those shown in Figure 2, we can confirm that the molecule has not been modified after being encapsulated and released from the produced nanoparticles. The UV-vis calibration curve (λ_max_ 485 nm) has been used for the quantification of encapsulated Lyc following dissolution of the same quantity of NPs. The Lyc@PLGA@PVA NPs and Lyc@PLGA@Tween 20 NPs have been loaded with 3.2 ± 0.29 µg/mg NPs and 2.1 ± 0.36 µg/mg NPs of Lyc, respectively. These data are obtained as the average of three independent synthesis batches. The PVA stabilization protocol results in a higher load of Lyc from the feeding TP extract.

**Figure 5:**
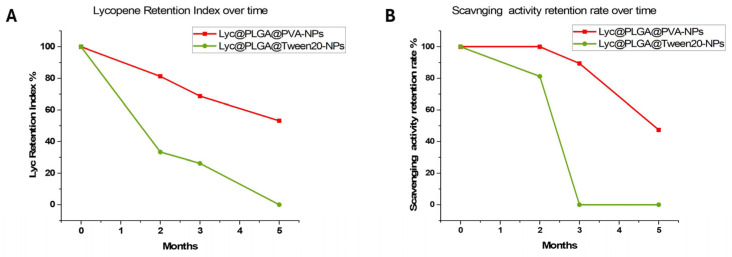
(**A**) Lyc retention index % and (**B**) scavenging activity retention rate % of freeze-dried Lyc@PLGA@PVA NPs and Lyc@PLGA@Tween 20 NPs over time, stored at 4 °C.

A UV-vis quantification assay has also been used to measure the Lyc retention index over time from Lyc@PLGA NP powder (at 4 °C). As we can observe in the plot of Figure 5A, PVA emulsifier provided a higher stability in terms of Lyc retention than Tween 20.

The Lyc@PLGA@PVA NP particles lose 40% of the encapsulated Lyc after 5 months of storage; on the other hand, the Lyc@PLGA@Tween 20 NPs lose all their content due to greater degradation of the compound during storage.

The stability of Lyc loaded into PLGA@PVA NPs has also been confirmed by a DPPH assay measuring the antioxidant activity retention percentage of Lyc solutions after Lyc@PLGA NP dissolution. In the plot of Figure 5B, we can observe a low loss of antioxidant activity after 5 months of storage for PLGA@PVA NPs. For the Lyc@PLGA@Tween 20 NPs, scavenging activity has been maintained after 2 months of storage despite the loss of ≅80% of Lyc retention. After three months of storage, the retained Lyc in the Tween 20 NPs was inactive under the investigated conditions.

Considering the measured Lyc retention over time by UV adsorption, samples that are more than one month old are not ideal for internalization studies using fluorescence, whether for PVA or Tween 20. Then, to facilitate the study of surfactants’ influence on Lyc@PLGA NPs’ interaction with spheroids, the nanoparticles used for biological testing were no older than one month.

### 3.3. Influence of Surfactants on Lyc@PLGA NPs’ Interaction with HaCaT and SK-MEL-2 Spheroids

#### 3.3.1. Cell Viability and Uptake of Lyc@PLGA NPs

MTT data, reported in Figure S6, indicated a low reduction in cell viability of HaCaT adherent cells after treatment with Lyc@PLGA@Tween 20 NPs at 200 µg/mL, that does not change over time (Figure S6A). SK-MEL-2 adherent cell viability has been affected by both NPs, with a linear decrease in percentages increasing Lyc@PLGA@Tween 20 NP concentrations over time (Figure S6B). Concerning Lyc@PLGA@PVA NPs, the toxic effect, revealed at 200 µg/mL after 48 h of treatment, does not persist after 72 h, suggesting a slowdown in cell proliferation (Figure S6B). MTT data on HaCaT and SK-MEL-2 spheroids have shown that both the produced NPs do not affect cell viability after 24 h of treatment at 100–200 µg/mL (Figure S6C,D). These NP concentrations have been selected for the subsequent biological assays.

**Figure 6:**
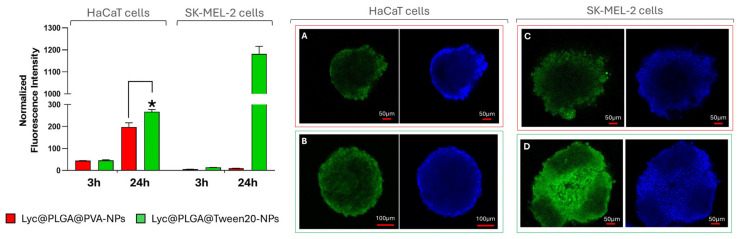
Uptake of Lyc@PLGA@PVA NPs and Lyc@PLGA@Tween 20 NPs in HaCaT and SK-MEL-2 spheroids by confocal imaging. On the left, intensity fluorescence (normalized for spheroid volume) measured after 3 and 24 h of treatment. Data have been reported as mean ± SD of 3 spheroids for each condition, analyzing 30 z stacks. Statistically significant value (*) *p* ≤ 0.05 versus Lyc@PLGA@PVA NP 24 h treatment, from the *t*-test. On the right, representative images of HaCaT and SK-MEL-2 spheroids after 24 h of treatment with Lyc@PLGA@PVA NPs (**A**,**C**) and Lyc@PLGA@Tween 20 NPs (**B**,**D**); in green, emitted fluorescence of Lyc (λ_exc._ = 488 nm), and in blue, cell nuclei stained with DAPI (λ_exc._ = 405 nm).

The normalized fluorescence intensities by confocal microscopy analysis of the treated spheroids are reported in Figure 6. Fluorescence intensity referred to Lyc excitation at 488 nm. Normalization was performed with respect to the volume of the spheroid that was analyzed. In Figure 6A–D, representative images of HaCaT and SK-MEL-2 spheroids after 24 h of treatment are reported.

HaCaT spheroids showed an increase in Lyc@PLGA NP internalization over time. As a function of time, there is a marked increase in normalized intensity, with a 4.5-fold rise observed for Lyc@PLGA@PVA NPs and with a ≅6-fold rise for Lyc@PLGA@Tween 20 NPs, from 3 to 24 h of treatment. The significant difference between Lyc@PLGA@PVA NPs and Lyc@PLGA@Tween 20 NPs at 24 h indicated an impact of surfactant on NP uptake in HaCaT spheroids.

SK-MEL-2 spheroids showed very low internalization for both investigated NPs after 3 h of incubation. Instead, a clear difference between the two surfactants has been observed after 24 h of incubation. Lyc@PLGA@PVA NP internalization remained at the low value that had been recorded at 3 h, instead of Lyc@PLGA@Tween 20 NP internalization, which considerably increased from 14.3 ± 0.26 to 1182 ± 34.4.

The gallery images reported in Figure S7 show the penetration of NPs across spheroids after 24 h of incubation. Concerning HaCaT spheroids, we observe that Lyc@PLGA@Tween 20 NPs were internalized more than Lyc@PLGA@PVA NPs, as measured by fluorescence quantitative analysis. Clearly, there is a greater accumulation of all particles along the periphery of the spheroid on the top, compared to a more homogeneous distribution on the bottom. Greater uniformity is observed for Lyc@PLGA@Tween 20 NPs penetration than Lyc@PLGA@PVA NPs (see Figure S7).

#### 3.3.2. Antioxidant Activity of Lyc@PLGA NPs

**Figure 7:**
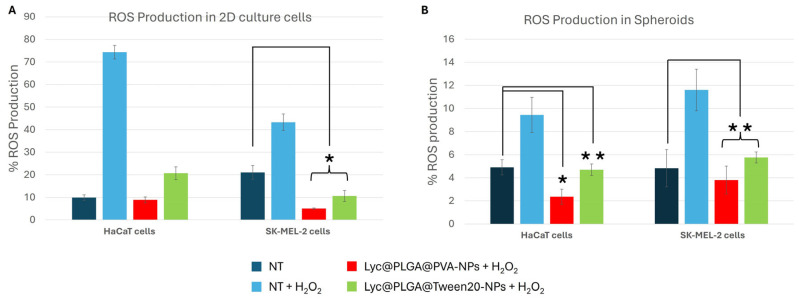
ROS production percentages in HaCaT and SK-MEL-2 2D culture (**A**) and HaCaT and SK-MEL-2 spheroids (**B**), reporting the negative control as the untreated condition (blue histograms), the positive control as treatment with only H_2_O_2_ (light blue histograms), and treated conditions as treatment with NPs and H_2_O_2_ (red and green histograms). (**A**) Data have been reported as the mean ± SD of 3 experiments; (**B**) data have been reported as the mean ± SD of 3 experiments, in which 12 spheroids were used for each condition. Statistically significant value (*) *p* ≤ 0.05 and (**) *p* ≤ 0.5 are versus the non-treated condition (blue histograms), from the *t*-test.

ROS production percentages measured with DCFDA assay are reported in Figure 7. HaCaT and SK-MEL-2 adherent cells and spheroids have been treated with 200 µg/mL of Lyc@PLGA@PVA NPs and Lyc@PLGA@Tween 20 NPs and H_2_O_2_ (1 mM) for 24 h. Representative flow cytometric spectra of intracellular ROS analysis are reported in Figure S8. The negative control is defined as the condition that has not been subjected to any treatment, while the positive control is defined as the condition that has been subjected to treatment with only H_2_O_2_.

The ROS inhibition effect of Lyc-loaded NPs is evident for both investigated samples, in 2D as well as in spheroids. Lycopene delivered by PLGA NPs restores ROS levels to those of the control condition, despite the oxidative stimulus of hydrogen peroxide. This effect has been detected both in 2D and spheroids. In SK-MEL-2 adherent cells, a significative ROS reduction with respect to the non-treated condition has been detected, following Lyc@PLGA@PVA NP and Lyc@PLGA@Twen 20 NP incubation (Figure 7A). This ROS reduction with respect to the non-treated condition has also been found in HaCaT and SK-MEL-2 spheroids (Figure 7B).

## 4. Discussion

The TP extract, obtained with SC-CO_2_, has been kindly provided by Licofarma s.r.l. (Galatina, Lecce, Italy) in the oil form. Many analytical methods have been used to assess Lyc isomers in food or extracts, such as UV–vis spectrophotometry and HPLC [21]. Solubilization of Lyc is quite difficult due to its high hydrophobic index. Here, we dissolved both commercial and TP extract in ethyl acetate, taking advantage of Lyc’s high solubility in this solvent (145.3 mg/mL), which is also used to synthesize PLGA nanoparticles. The chromatographic profile of TP extract corresponds to that of the standard in the same solvent (ethyl acetate) and HPLC conditions. The compounds are separated by their Rt values, which, for the all-trans-lycopene standard and its isomers, are in the range of 8–20 min, using C18 and C30 columns [21]. The major compound identified in the extract was Lyc at Rt 9.91 min, consistent with the identified peak of commercial Lyc (see Figure 1).

UV–vis spectrophotometry has been employed for the simple and rapid detection of Lyc in TP extract, comparing with the commercial standard. The UV-vis spectrum of Lyc samples in organic solvents reported different maximum absorption wavelengths depending on the different solubilization methods [21]. Scanning the region 350–550 nm, three maximum absorbance bands have been revealed at λ_max_ 457, 482, and 513 nm [31,53], similar to those obtained for the tomato Lyc standard (λ_max_ 460, 485, and 516 nm); see Figure 2. The measured Abs at λ_max_ 485 nm has been chosen for the quantification experiments on Lyc@PLGA NPs.

The DPPH assay has confirmed the great antioxidant activity of TP extract thanks to the comparison with the same quantity of ascorbic acid, used as a positive control (96% of inhibition for both samples). The chemical nature of these compounds is different; Lyc is a hydrophobic molecule, and ascorbic acid is a water-soluble vitamin. Despite this difference, they are important nutrients found in fruit and vegetables (particularly tomatoes), and they work together to promote cellular health, reducing inflammation, supporting the immune system, and protecting skin from oxidative stresses. Lipid-soluble Lyc acts within cell membranes, while water-soluble ascorbic acid acts within the cytoplasm, although they work together to neutralize ROS and regenerate other antioxidants, such as vitamin E. The dose-dependence assay enabled us to evaluate the effect of extract concentration on the DPPH protocol and determine the concentration at which almost 100% inhibition was achieved. The high scavenging activity of TP extract resulted in dose dependence; this data is important given that the TP extract is in oil form. The linearity of in vitro antioxidant activity excludes that the solubilization of a given amount of Lyc contained in the extract can be affected by heterogeneity of lipid matrix.

Having verified the presence of Lyc in the initial extract and determined the optimal conditions for its characterization and quantification, we proceeded to focus on its efficient encapsulation.

PLGA is a copolymer approved by the U.S. FDA and is currently used in a broad range of biomedical applications thanks to its biocompatibility, biodegradability, and capacity to preserve loaded drug degradation [58]. Lyc@PLGA NPs have been fabricated through the double emulsion method using ethyl acetate as the organic solvent and a polymer/TP extract ratio of 2/1. The effects of polymer concentration, the type of organic solvent used, temperature, and the injection and agitation rates of the organic and aqueous phases were widely studied [59]. However, surfactants play a key role in the stabilization phase of polymeric NP emulsion formation, in their size, and finally, in biological interactions [45,46,47].

We have compared two non-ionic and biocompatible surfactants: Tween 20 and PVA. Tween 20 is the commercial name for polysorbate 20, a type of emulsifier utilized in food and pharmaceutical fields, as well as cosmetics, particularly in products for the skin, hair, and nails. PVA offers interesting versatility for a variety of industrial applications, including controlled drug delivery systems, tissue adhesion barriers, transdermal patches, and tissue engineering [48]. The deposition of non-ionic surfactants at the interface of emulsified w/o droplets affected their size, and therefore affected the final colloidal stability and PdI. The average hydrodynamic diameter (see Table 1) and the size distribution by intensity (see Figure S2) suggest that Tween 20 has a greater stabilizing effect. The smaller Z-average (Table 1) of the Lyc@PLGA@Tween 20 NPs means there is less aggregation of nanoparticles in solution than with PVA. Under SEM observation, the actual sizes of Lyc@PLGA@PVA NPs and Lyc@PLGA@Tween 20 NPs are very similar (see Figure 3 and Figure S3). The colloidal stability in water has also been confirmed by ζ-potential measurements, which differed between the two surfactants. Nanoparticles acquire a surface charge of ≅−44 mV with Tween 20, compared to ≅−30 mV with PVA (Table 1). Both NPs maintained this negative charge after 25 days in suspension (Table S2), suggesting a stabilization effect of carriers in liquid media over time. The more negative ζ-potential value of Lyc@PLGA@Tween 20 NPs confirmed their improved distribution in size, since this parameter is recognized as an indicator of colloidal stability. When the surface charge of NPs is homogeneous (i.e., either positive or negative), the Van der Waals and electrostatic forces are known to oppose each other, leading to the formation of a stable suspension, without flocculation or precipitation [60]. PLGA nanoparticles are typically negatively charged due to the carboxyl groups on the polymer surface, with a ζ-potential often around −20 mV, as confirmed by our measurements (see Table S1). As both PVA and Tween 20 are non-ionic, the interpretation of the ζ-potential in order to predict the stability of our colloidal nanosuspensions is linked to the resulting surfactants’ steric effect [48]. Furthermore, considering the DLS parameters values for empty NPs, we can deduce that the adsorption of these hydrophilic emulsifiers on Lyc@PLGA NPs reduced their hydrophobicity and decreased the interfacial tension between Lyc and PLGA, leading to relatively homogeneous and stable nanosuspensions. Our data suggested that Tween 20’s emulsification capability is superior to that of PVA, concerning Lyc-loaded NPs. This result is not in accordance with previous reports [61,62], suggesting that the interaction with the loaded compound could also play a crucial role. A previous study on Lyc-loaded polysaccharide NPs showed that PVA concentration significantly impacts colloidal stability and molecule loading [63]. The high hydrophilic–lipophilic balance and low critical micelle concentration of polysorbates give Tween 20, when used, high surface activity, even at low concentrations (the protocol provided for 0.5% *w/v* of both surfactants). Instead, PVA stabilizes emulsions by forming an interconnected network with the polymer at the interface (through hydrogen bond formation), resulting in a fraction of PVA remaining associated on the NPs [48,64]. This feature can be observed by comparing the morphological structure of the synthesized NPs. Thin ridges have been primarily observed on the surface of Lyc@PLGA@PVA NPs (see Figure 3), suggesting the presence of an emulsifier.

Material choice, including emulsifiers, affect particle size, zeta potential, surface morphology features, and also drug loading [48,49]. Lycopene loading has been verified through FT-IR analysis on freeze-dried NPs and on Lyc solution after NP dissolution. The FT-IR analysis offered a qualitative indication of Lyc encapsulation within the PLGA nanoparticles. As shown in Figure 4, the spectra of the loaded formulations were dominated by the characteristic bands of the polymer matrix, and FT-IR therefore could not provide quantitative information on Lyc content. Although the overall spectral profile of both Lyc@PLGA NPs resembles that of blank PLGA due to the predominance of the polymer matrix, marked differences emerge in the highlighted regions, confirming the contribution of the encapsulated Lyc [65,66,67]. The attenuation and partial overlap of these signals can be attributed to matrix effects and to the solid-state nature of the formulations, a behaviour commonly observed for carotenoids embedded in polymeric carriers [68,69]. Despite these intrinsic limitations, the subtle yet reproducible spectral differences detected in the relevant regions of Figure 4 were sufficient to qualitatively confirm encapsulation. The analysis performed after NP dissolution (Figure S4) further reinforced these observations. Under homogeneous solvent conditions, the vibrational profile of the released Lyc matched that of the TP extract, suggesting that the encapsulation and release processes did not cause detectable alterations to the characteristic polyene structure. The persistence of the characteristic band at 960 cm^−1^ confirms that the polyene chain remained intact, and that the carotenoid preserved its structural configuration throughout the formulation and release processes [67,70]. Although qualitative, this agreement supports the conclusion that the molecule maintains its structural integrity throughout the process. Overall, FT-IR offered qualitative confirmation of Lyc encapsulation and complemented the quantitative loading values obtained through UV–vis analysis.

UV-vis analysis has been used to quantify loaded Lyc; the calculated value of µg Lyc/mg NPs indicates a higher compound content for Lyc@PLGA@PVA NPs. Larger particles commonly have a higher drug loading capacity; moreover, PVA has been widely demonstrated to be highly efficient in synthesizing carriers for the effective loading of different drugs [48,71].

In addition, emulsifiers, such as polysorbate and PVA, are also known to confer stability to NPs during storage in an aqueous medium and/or after freeze-drying. The colloidal stability of Lyc@PLGA NP aqueous suspensions has been discussed relating to DLS parameters. In view of the unstable nature of the compound, a series of tests were also carried out to assess the stability of loaded Lyc over time. These tests evaluated the retention and antioxidant activity of Lyc, following NP powder dissolution after storage. The protective effect of PLGA on Lyc has previously been demonstrated in a simulated gastrointestinal tract system [44]. Emulsifiers could also play a key role in retaining and stabilizing Lyc [72]. Retention index and scavenging activity percentages suggested a better capacity to protect Lyc toward degradation for Lyc@PLGA@PVA NPs with respect to Lyc@PLGA@Tween 20 NPs, in accordance with the known strong interaction of PVA with the NP surface [48]. There is a linear tendency between both measured indexes and storage time for Lyc@PLGA@PVA NPs. Regarding the Lyc@PLGA@Tween 20 NPs, the scavenging activity remained after two months of storage, despite a considerable loss of Lyc. This is probably due to isomers forming as a result of Lyc degradation; these isomers nevertheless remain bioactive [6,19]. Our results confirmed the strategic role of nanoparticles, comparing with microencapsulation and electrospinning techniques which provide a Lyc retention rate of 30–50% after ≅30 days of storage at room temperature, and ≅40% after 14 days at 4 °C [18,73]. The delayed degradation that has been observed, especially for Lyc@PLGA@PVA NPs, is comparable to that found for other nanocarriers [30,72,74].

Our primary objective was to compare the influence of two non-ionic surfactants (PVA and Tween 20) on NP performance. Crucially, we relied on Lyc’s intrinsic emitted fluorescence to monitor internalization in human spheroids, deliberately avoiding external probes that could alter the physicochemical properties of the carriers. Considering the measured Lyc retention over time, samples older than one month exhibit reduced UV absorption, rendering them unsuitable for accurate internalization studies. Consequently, to disentangle storage-related degradation from the surfactant’s actual impact on cellular interaction, and to ensure comparable PLGA NPs properties, we performed biological assays using samples within a defined post-synthesis timeframe (≤1 month).

Furthermore, the performed stability assays on NP aqueous suspensions and stored powder consist of a preliminary screening to support future dermatologic formulation and shelf-life validation.

In this context, the evaluation of surfactant-induced stability in an efficient biological environment represents a fundamental rational basis for the study. Indeed, ensuring that the Lyc remains encapsulated and protected from rapid degradation is a mandatory prerequisite; without proof of such foundational stability and antioxidant retention, further complex investigations, such as Franz diffusion cell experiments, would lack a scientifically sound starting point.

The biological interactions of NPs are contingent on their size and surface phenomena. Consequently, the cellular uptake and bioactivity of delivery systems are also dependent on the surfactants used [46,47]. Their hydrophilic nature promotes interaction with cells, ligands, and proteins, which may lead to benefits as well as potential toxic effects that need to be investigated [48].

First, we studied cell availability through MTT tests treating HaCaT and SKMEL-2, 2D and 3D cultures, with different concentrations of Lyc@PLGA@PVA NPs and Lyc@PLGA@Tween 20 NPs. MTT data (see Figure S6) suggested that 100 µg/mL and 200 µg/mL of Lyc@PLGA@ NPs could safely be used to treat cells for 24 h.

The skin is a complex organ composed of multiple layers. The most superficial layer is the epidermis, which represents the main physical and chemical barrier to permeation by bioactive agents and NPs. Keratinocytes are the most abundant cell type in the epidermis, accounting for 90% of cells. They are responsible for producing keratin, the organ’s major physical barrier [75]. It has been established that NPs are able to penetrate the skin via the same pathways that have been described for other substances. However, it is important to note that the ability of NPs to penetrate varies according to their size, composition, and colloidal stability [75]. For example, negatively charged NPs cross the epidermis more easily than those with a positive charge [76]. Polymeric NPs can cross the epidermis by penetrating the stratum corneum, accumulating on the upper surface, and penetrating the hair follicle (where melanocytes are located) [75].

There are a few problems with using 2D human keratinocytes and melanocytes as a replacement for animal experiments. The main problems are that they do not replicate the in vivo complexity, the cellular architecture comprising the extracellular matrix microenvironment, and cell–cell interactions. Three-dimensional cell culture systems overcome many of these limitations, more closely mimicking the complex phenotypic heterogeneity produced during cell growth [50,77]. The merits of in vitro skin 3D models extend to the field of cosmetics development. Regarding skin applications, the utilization of animal models frequently proves to be suboptimal. It is a commonly held view that economically advantageous species, which include rodents, have a skin composition that is too dissimilar to human skin. Conversely, animals with more human-like skin, such as pigs, are too difficult to manage and too expensive for regular use. Moreover, ethical considerations further underscore the limitations of animal models in skin research.

We investigated the uptake and penetration of Lyc@PLGA NPs across spheroids of HaCaT and SK-MEL-2 cells, to ascertain the function of PLGA NPs and the impact of surfactants. The employment of spheroids is a very interesting tool due to their simplicity, low cost, and high reproducibility. Hence, they are suitable for high-throughput cell function and cytotoxicity analysis, biochemical analysis, and different imaging techniques, including confocal microscopy [50]. Moreover, our analysis exploited the emitted fluorescence of Lyc. This avoids the limitations associated with fluorochrome labelling of NPs, which could interfere with their physicochemical properties and mask the impact of surfactants. This assay is a critical step that precedes time-consuming and costly ex vivo testing and justifies it.

The internalization of the Lyc@PLGA@Tween 20 NPs after a 24 h treatment period is observed to be greater in comparison to that of the other NPs. This phenomenon is probably attributable to the markedly negative ζ-potential exhibited by the Lyc@PLGA@Tween 20 NPs, in conjunction with their comparatively low average hydrodynamic diameter [75]. Furthermore, the greater colloidal stability of Lyc@PLGA@Tween 20 NPs in aqueous solution can encourage interaction with cells, promoting their internalization at the same treatment time and concentration. This difference is even greater when SKMEL-2 cells are compared to HaCaT cells; this is probably due to the increased phagocytic activity of the cancer cells. A comparatively low proportion of Lyc@PLGA@PVA NPs are internalized in SK-MEL-2 cells in comparison to Lyc@PLGA@Tween 20 NPs (see Figure 6). Thus, Tween 20 promotes the NPs’ uptake by melanocytes, providing a potential exploitation of this surfactant for anticancer drug delivery, also considering the detected moderate toxic effect (see Figure S6). It has been demonstrated through in vitro studies that the chemical composition of nanocarriers, in conjunction with the presence of emulsifiers, exerts a significant influence on cell uptake and penetration [23,78]. However, the distinguishing feature of our study is the in vitro assay on cell spheroids with confocal analysis, which exploits the intrinsic fluorescence of the loaded compound. Furthermore, it can be deduced from the observation of the Z-stacks in Figure S7 that the NPs are capable of homogeneous penetration across HaCaT and SK-MEL-2 spheroids. This assay constitutes a proof-of-concept investigation into the role of the produced carriers in terms of their uptake by skin cells, which are organized in a 3D architecture that is useful in simulating the complex in vivo environment. It is anticipated that the results of the present study will be utilized in future transdermal studies, with a view to validating the Lyc penetration and distribution.

Despite the divergent internalization of the produced NPs by the surfactant employed, the loaded Lyc exhibited remarkable antioxidant activity, as evidenced by its capacity to inhibit intracellular ROS production following treatment with Lyc@PLGA NPs. As illustrated in Figure 7, both Lyc@PLGA@PVA NPs and Lyc@PLGA@Tween 20 NPs were found to restore the ROS levels to those of the non-treated cells, thereby avoiding the oxidative stimulus of H_2_O_2_ treatment. This outcome is in accordance with the findings of previous studies on Lyc-loaded carriers [79].

## 5. Limitations of the Study and Future Perspectives

We have developed a protocol for the nanoencapsulation of Lyc from tomato extract in PLGA nanoparticles, and have studied their antioxidant activity in human skin spheroids. We fully recognize the importance of ex vivo skin permeation data, such as those obtained via Franz diffusion cells, for the validation of dermatological formulations. However, the present study was specifically designed as a foundational proof-of-concept and a rational screening phase. Our primary objective was to elucidate how the choice of surfactants dictates the physicochemical stability, antioxidant retention, and biological performance of Lyc@PLGA NPs. The significance of surfactants and the wide range of their functions have been extensively debated. By employing 3D human skin spheroids, we have provided a significantly more sophisticated and physiologically relevant assessment of cellular uptake and ROS inhibition than traditional 2D cultures, as previously discussed. This approach serves as a necessary methodological bridge, establishing the essential biological safety and efficacy parameters before proceeding to costly ex vivo trials. The findings presented here provide a rational basis for selecting Lyc@PLGA@PVA NPs as the lead candidate for advanced development. Future investigations will involve the integration of these optimized nanosystems into complete topical vehicles, followed by long-term stability protocols and quantitative permeation studies using ex vivo human skin to fully validate their translational and industrial potential.

Lycopene has been selected as a challenging model compound due to its interesting biological properties. However, Lyc is not among the most potent antioxidants currently available; there are several alternative compounds, both natural and synthetic, exhibiting significantly higher antioxidant capacity while also offering superior chemical stability. This choice was also influenced by selected sources for molecule extraction. The Lyc-rich extract has been supplied by Licofarma s.r.l. in oil form, which was obtained using a SC-CO_2_ process on tomato peels. Licofarma s.r.l. holds a patent for extracting Lyc from tomatoes using SC-CO_2_ and uses the resulting extract as an ingredient in different cosmetic/nutraceutical formulations. We worked to efficiently synthesize Lyc-loaded NPs as an alternative cosmetic ingredient to oily SC-CO_2_ TP extract. The exploitation of tomato peel by Licofarma s.r.l. addressed the increasing trend of utilizing waste recovery as prospective sources of bioactive compounds. So, the choice of Lyc as a model compound is also motivated by the challenge of implementing sustainable, circular economy practices. Furthermore, the investigated protocols and analytical methods will be a platform for other bioactive molecule encapsulations with different industrial relevance, including the utilization of other natural or waste sources.

Concerning physicochemical stability, DLS measurements demonstrated that the tested surfactants were capable of yielding Lyc-loaded NPs that exhibited stability within an aqueous solution over a period of up to 25 days. However, the absence of a long-term stability assay on NP suspensions is a limit of the proposed investigation.

Once the NPs have been synthesized, they must be incorporated into a vehicle suitable for dermatologic use. Prior to being incorporated into a cosmetic formulation, NPs are usually characterized in terms of their morphology, size distribution, zeta potential, and encapsulation and/or loading efficiency. We have to consider that the physical stability of skincare formulations containing NPs depends not only on the nature of the interactions between nanoparticles, but also on their interaction with the dispersant and emulsifiers. In this scenario, our proof-of-concept study into the role of surfactants impact is pivotal, working as a screening tool.

Our main future focus is the development of suitable skincare formulations based on Lyc@PLGA NPs. Moreover, the selection of an appropriate vehicle is contingent upon the intended purpose of the cosmetic product. Therefore, we will prepare the potential skincare formulation by adding small volumes of nanosuspension directly to a gel, cream, or o/w emulsion, mixing them together to obtain the final product. Some precautions should be observed: the appropriate concentrations of surfactants or polymers must be determined to prevent the particles from growing and aggregating during storage; the use of ionized additives such as electrolytes should also be avoided to prevent nanocolloid agglomeration. Finally, the product’s evaluation will include physical and chemical analysis, monitoring organoleptic properties, colour, odour, the presence of phase separation, pH, and viscosity. The potential Lyc release rate from the formulation will also be analyzed exploiting in vitro assays (e.g., systems like Franz cells).

The preliminary storage stability test indicated that surfactants play a critical role in maintaining the retention and activity of the encapsulated Lyc. Freeze-dried Lyc@PLGA@PVA NPs have a good stability up to 5 months of storage at 4 °C; instead, Lyc@PLGA@Tween 20 NPs remain fairly stable after 2 months of storage at 4 °C. This is a limitation that will be considered in future studies, which will need to take into account the storage time based on the surfactant used for the synthesis of the nanoparticles, the storage temperature, and the samples’ state (suspended in water, wet, or dry).

Shelf lives of at least 12–24 months are generally required for cosmetic or pharmaceutical ingredients. Therefore, a shelf-life evaluation will be performed under different temperatures, extending the time beyond five months, to understand the stability of the skincare formulation based on Lyc@PLGA NPs.

Our proof-of-concept study has been preceded with biological assays verifying the cytocompatibility, cellular uptake efficiency, and antioxidant activity of Lyc-loaded NPs.

Transdermal penetration evaluation is crucial for potential active ingredients and carriers because, as previously discussed, the skin is a very selective organ.

We conducted a preliminary cellular uptake study to investigate the influence of surfactants on the efficient internalization of Lyc in human keratinocytes and melanocytes, which were selected as cellular models of the epidermis. We performed confocal microscopy to provide z-stack imaging and quantify fluorescence following the treatment of human skin spheroids with NPs. Furthermore, since Lyc is fluorescent, we were able to visualize its uptake directly in the acquired z-stacks, avoiding NP fluorochrome labelling. Our results suggest that Lyc@PLGA@Tween 20 NPs enhance Lyc uptake in spheroids, particularly in melanocytes. This indicates that Tween 20 plays a crucial role in promoting cell interaction.

It is evident that the available data is inadequate for the purpose of substantiating claims pertaining to the penetration, retention, and transport of Lyc across the skin layers.

The choice to prioritize the study of surfactant-cell interactions via 3D models serves to validate the performance of the formulation at the cellular level, bypassing the mentioned limits of 2D cell cultures. This screening is crucial: confirming that NPs are stable enough to deliver their cargo and exert a biological effect within a stratified cellular architecture provides the necessary justification for future, more resource-intensive ex vivo permeation trials. While the cellular spheroids provide valuable insights into cellular internalization and antioxidant protection within their 3D structure, it does not account for the barrier function of the stratum corneum. Future studies employing Franz diffusion cells and ex vivo skin models are already planned to quantify the actual permeation performance.

However, the spheroid uptake assay will be employed in future investigations involving new spheroids generated from co-cultures, which are designed to better mimic the human epidermis and provide a more efficient ex vivo model. We also plan to use commercially available human epidermis models to address the challenge of conducting animal-free studies.

Although, the antioxidant activity in terms of ROS inhibition following oxidative stress is great for both studied samples. This means that the surfactant affected NP internalization in spheroids without interfering with bioactivity, which remains high even at lower levels of internalized Lyc. This is undoubtedly due to Lyc’s significant intrinsic antioxidant capacity and increased bioavailability from carriers.

Lyc@PLGA NP-enriched formulations will be tested for long-term and transdermal permeation, verifying the antioxidant activity in human spheroids as well as in a commercial reconstructed human epidermis. Proteomics and metabolomics will be performed to understand the biochemical pathways triggered by Lyc released in target cells (keratinocytes or melanocytes) to support the preliminary biological data on 2D and 3D cell cultures and to exclude toxicity and/or side effects of nano-formulations. The omics sciences offer technological advances that are well-integrated into the concept of “cosmeceuticals”, which refers to cosmetics that have a scientifically proven preventive or curative effect.

These future perspectives will explore the data of the present proof-of-concept study and support the industrial relevance of potential skincare formulations based on Lyc@PLGA NPs.

## 6. Conclusions

The present study is a proof-of-concept investigation into the effect of surfactants on the properties of PLGA NPs, using Lyc as a challenging model compound. We have compared PVA and Tween 20 as surfactants for colloid stabilization and efficient Lyc loading. Colloidal stability in aqueous medium is affected by the surfactant, as demonstrated by the better DLS parameters of Lyc@PLGA@Tween 20 NP suspensions with respect to Lyc@PLGA@PVA NPs. The stability of loaded Lyc retention and scavenging capacity over time was enhanced by Lyc@PLGA@PVA NPs, suggesting a protective effect from PVA. Chemical assays provide a controlled and reproducible means of monitoring the loss of radical scavenging capacity due to degradation during storage, which is a critical parameter for skincare formulation and shelf-life evaluation. The concentrations and treatment times were selected based on the stability and cytotoxicity data to investigate Lyc@PLGA NPs’ effect on cell uptake and bioactivity. We performed a cell uptake assay on HaCaT and SK-MEL-2 spheroids, measuring the fluorescence of encapsulated Lyc directly using confocal imaging. This study emphasized the pivotal role of Tween 20 in facilitating the internalization of NPs by human melanocytes, thereby underscoring its potential for further applications. Despite the detected effect on cell uptake, the bioactivity of Lyc is enhanced by delivery via all Lyc@PLGA NPs. The substantial antioxidant capacity of Lyc@PLGA@PVA NPs and Lyc@PLGA@Tween 20 NPs in HaCaT and SK-MEL-2 spheroids could offer significant potential for skin protection. The data obtained on stability and bioactivity will be exploited for future performance research on effective skincare formulations containing the nanosized ingredients. Biological assays will be conducted to verify cytocompatibility, transdermal penetration/distribution, and ROS inhibition. Moreover, the stability of loaded Lyc in a cellular environment will be investigated by testing the final formulations.

Future perspectives provide long-term stability tests and sophisticate transdermal permeation studies to support the industrial relevance of Lyc@PLGA NP-based skincare formulations.

## Figures and Tables

**Table 1:** DLS parameters measured on diluted Lyc@PLGA NP suspensions in filtered distilled water (0.45 μm).

Sample	ζ-Potential (mV) ^1^	Z-Average (nm) ^2^	PdI ^3^
Lyc@PLGA@PVA NPs	−28.6 ± 1.15	393.5 ± 25.19	0.44 ± 0.021
Lyc@PLGA@Tween 20 NPs	−43.6 ± 1.47	177.6 ± 8	0.4 ± 0.016

^1^ ζ-potential values are reported as the mean (±standard deviation) of 5 measurements; each of them is derived from 20 different runs; ^2^ Z-average (±standard deviation) is the average hydrodynamic diameter of 3 distribution size measurements, and each one consists of 12 runs; ^3^ polydispersity Index (PdI) is reported as the mean (±standard deviation) of 3 distribution size measurements, and each one consists of 12 runs.

## Data Availability

The original contributions presented in this study are included in the article/Supplementary Materials. Further inquiries can be directed to the corresponding authors.

## Supplementary Materials

The following supporting information can be downloaded at: https://www.mdpi.com/article/10.3390/antiox15010136/s1. Figure S1. Scavenging activity percentage by DPPH inhibition assay versus TP extract mg; Figure S2. Size distribution plot as Intensity (%) of diluted Lyc@PLGA NP suspensions; Table S1. DLS parameters measured on empty PLGA-NP suspensions; Table S2. DLS parameters measured on Lyc@PLGA NP suspensions after 25 days; Figure S3. Representative SEM images of Lyc@PLGA@PVA NPs and Lyc@PLGA@Tween 20 NPs; Figure S4. FT-IR of Lyc solution after Lyc@PLGA NP dissolution; Figure S5. UV-vis spectra of Lyc solution after Lyc@PLGA NP dissolution; Figure S6. Cell viability data on HaCaT and SK-MEL-2 2D and Spheroids; Figure S7. Confocal microscopy gallery images; Figure S8. Representative flow cytometric spectra of intracellular ROS analysis.

## Abbreviations

The following abbreviations are used in this manuscript:

Lyc	Lycopene
PLGA	Poly-lactic-co-glycolic acid
PLGA NPs	PLGA nanoparticles
PVA	Polyvinyl alcohol
Lyc@PLGA NPs	Lycopene loaded PLGA nanoparticles
Lyc@PLGA@PVA NPs	Lycopene loaded PLGA nanoparticles with PVA
Lyc@PLGA@Tween 20 NPs	Lycopene loaded PLGA nanoparticles with Tween 20
SC-CO_2_	Supercritical CO_2_
TP	Tomato peel
