# Supplementary material for: Shaping Lycopene Nanoparticles Performance: How Surfactants Influence Stability, Antioxidant Activity, and Uptake in Human Skin Spheroids
Source: Antioxidants (Basel). 2026 Jan 21;15(1):136. doi: 10.3390/antiox15010136 (PMC12837230; doi:10.3390/antiox15010136)
Supplement: Supplementary file 1 [file antioxidants-15-00136-s001.zip › antioxidants-4056636-supplementary.pdf]

Shaping Lycopene Nanoparticles Performance: How Surfactants Influence Stability, Antioxidant Activity, and Uptake in Human Skin Spheroids

Francesca Baldassarre [1,2*], Chiara Boncristiani [3], Michela Ottolini [1], Concetta Nobile [4], Maryam Shahzad Shirazi [1], Giuseppe E. De Benedetto [5], Gianpiero Colangelo [6], Viviana Vergaro [3*], Ludovico Valli [1,2] and Giuseppe Ciccarella [1,2,4]

1 Department of Biological and Environmental Sciences and Technologies (DiSTeBA), University of Salento S.P. 6 Lecce-Monteroni SNC, 73100 Lecce, Italy; francesca.baldassarre@unisalento.it, michela.ottolini@unisalento.it, maryam.shahzadshirazi@unisalento.it, ludovico.valli@unisalento.it, giuseppe.ciccarella@unisalento.it
2 UdR INSTM, Salento, 73100 Lecce, Italy; francesca.baldassarre@unisalento.it, ludovico.valli@unisalento.it, giuseppe.ciccarella@unisalento.it
3 Department of Experimental Medicine, University of Salento, S.P. 6 Lecce-Monteroni SNC, 73100 Lecce, Italia; chiara.boncristiani@unisalento.it, viviana.vergaro@unisalento.it
4 Institute of Nanotechnology, CNR NANOTEC, Consiglio Nazionale Delle Ricerche, S.P. 6 Lecce-Monteroni SNC, 73100 Lecce, Italy; concetta.nobile@nanotec.cnr.it, giuseppe.ciccarella@unisalento.it
5 Department of Cultural Heritage, University of Salento, Via D. Birago 64, Lecce, 73100, Italy; giuseppe.debenedetto@unisalento.it
6 Department of Engineering for Innovation, University of Salento, S.P. 6 Lecce-Monteroni SNC, 73100 Lecce, Italia; gianpiero.colangelo@unisalento.it
* Correspondence: francesca.baldassarre@unisalento.it, F.B:; viviana.vergaro@unisalento.it, V.V.

Supplementary Materials

Summary

Supplementary Figure S1. Scavenging activity percentage by DPPH inhibition assay *versus* TP extract mg
Supplementary Figure S2. Size distribution plot as Intensity (%) of diluted Lyc@PLGA-NPs suspensions
Supplementary Table S1. DLS parameters measured on empty PLGA-NPs suspensions
Supplementary Table S2. DLS parameters measured on Lyc@PLGA-NPs suspensions after 25 days
Supplementary Figure S3. Representative SEM images of Lyc@PLGA@PVA-NPs and Lyc@PLGA@Tween20-NPs
Supplementary Figure S4. FT-IR of Lyc solution after Lyc@PLGA-NPs dissolution
Supplementary Figure S5. UV-vis spectra of Lyc solution after Lyc@PLGA-NPs dissolution
Supplementary Figure S6. Cells viability data on HaCaT and SK-MEL-2 2D and Spheroids
Supplementary Figure S7. Confocal microscopy gallery images
Supplementary Figure S8. Representative flow cytometric spectra of intracellular ROS analysis

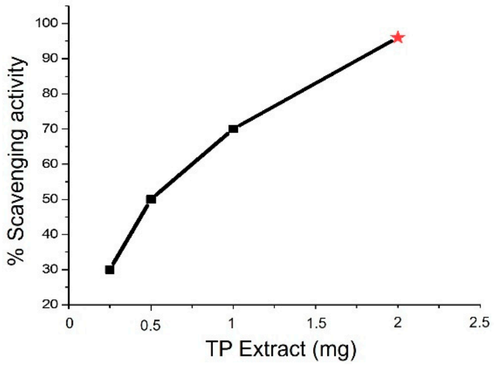

Figure S1. Scavenging activity percentage by DPPH inhibition assay *versus* TP extract mg, following solubilization in ethyl acetate. The red star indicated the corresponding maximum activity recorded also for the same quantity of the positive control, ascorbic acid.

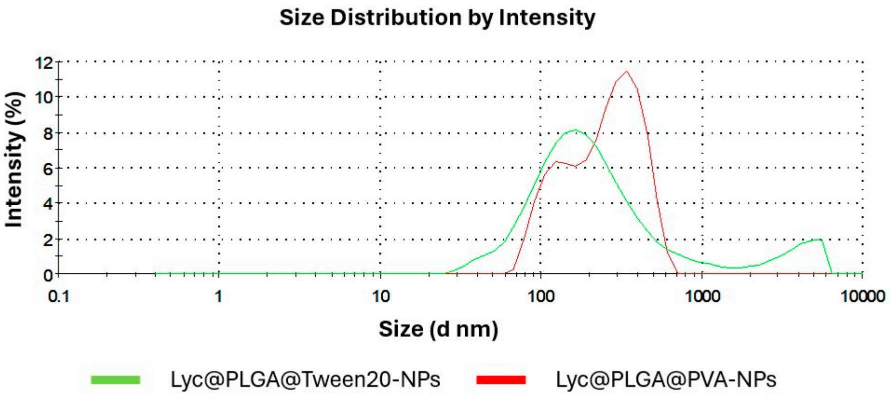

Figure S2. Size distribution plot as Intensity (%) of diluted Lyc@PLGA-NPs suspensions in filtered distilled water (0.45μm), after synthesis.

Table S1. DLS parameters measured on empty PLGA-NPs suspensions, diluted in filtered distilled water (0.45μm).

Sample	ζ-potential (mV)[1]	Z-average (nm)[2]	PdI[3]
PLGA@PVA-NPs	-18.7±1.4	255±3.2	0.18±0.006
PLGA@Tween20-NPs	-29.1±0.33	286±5.4	0.34±0.037

[1] ζ-potential values are reported as the mean (±standard deviation) of 5 measurements; each of them derived from 20 different runs; [2] Z-average (±standard deviation) is the average hydrodynamic diameter is the of 3 Distribution Size measurements, each one consists of 12 runs; [3] Polydispersity Index (PdI) is reported as the mean (±standard deviation) of 3 Distribution Size measurements, each one consists of 12 runs.

Table S2. DLS parameters measured on Lyc@PLGA-NPs suspensions after 25 days in filtered distilled water (0.45µm).

Sample	ζ-potential (mV)[1]	Z-average (nm)[2]	PdI[3]
Lyc@PLGA@PVA-NPs	-30.3±1.38	308±17.6	0.46±0.044
Lyc@PLGA@Tween20-NPs	-52.8±1.5	196,2±23.7	0.48±0.072

[1] ζ-potential values are reported as the mean (±standard deviation) of 5 measurements; each of them derived from 20 different runs; [2] Z-average (±standard deviation) is the average hydrodynamic diameter is the of 3 Distribution Size measurements, each one consists of 12 runs; [3] Polydispersity Index (PdI) is reported as the mean (±standard deviation) of 3 Distribution Size measurements, each one consists of 12 runs.

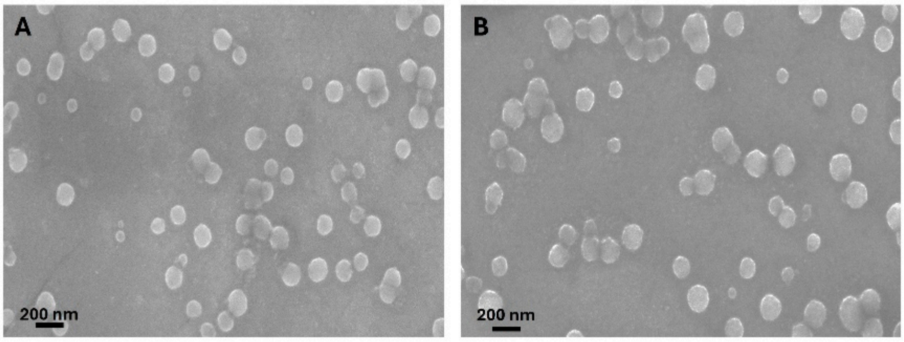

Figure S3. Representative SEM images at the same magnification of (A) Lyc@PLGA@PVA-NPs and (B) Lyc@PLGA@Tween 20 -NPs.

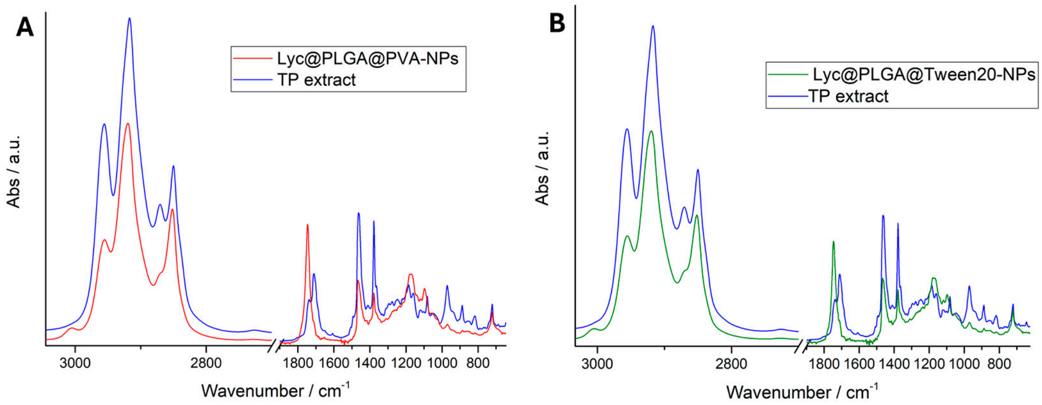

Figure S4. FT-IR spectra of Lyc solution following dissolution of 2 mg in 1 mL ethyl acetate for each sample, (A) Lyc@PLGA@PVA-NPs and (B) Lyc@PLGA@Tween20-NPs; each compared with the TP extract in ethyl acetate (blue line).

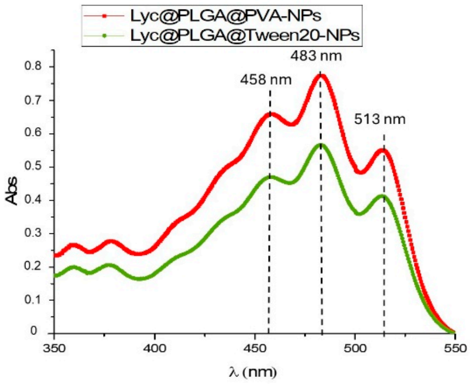

Figure S5. UV-vis spectra of Lyc solution following dissolution of 2 mg in 1 mL ethyl acetate for each sample, Lyc@PLGA@PVA-NPs and Lyc@PLGA@Tween 20 -NPs, after synthesis.

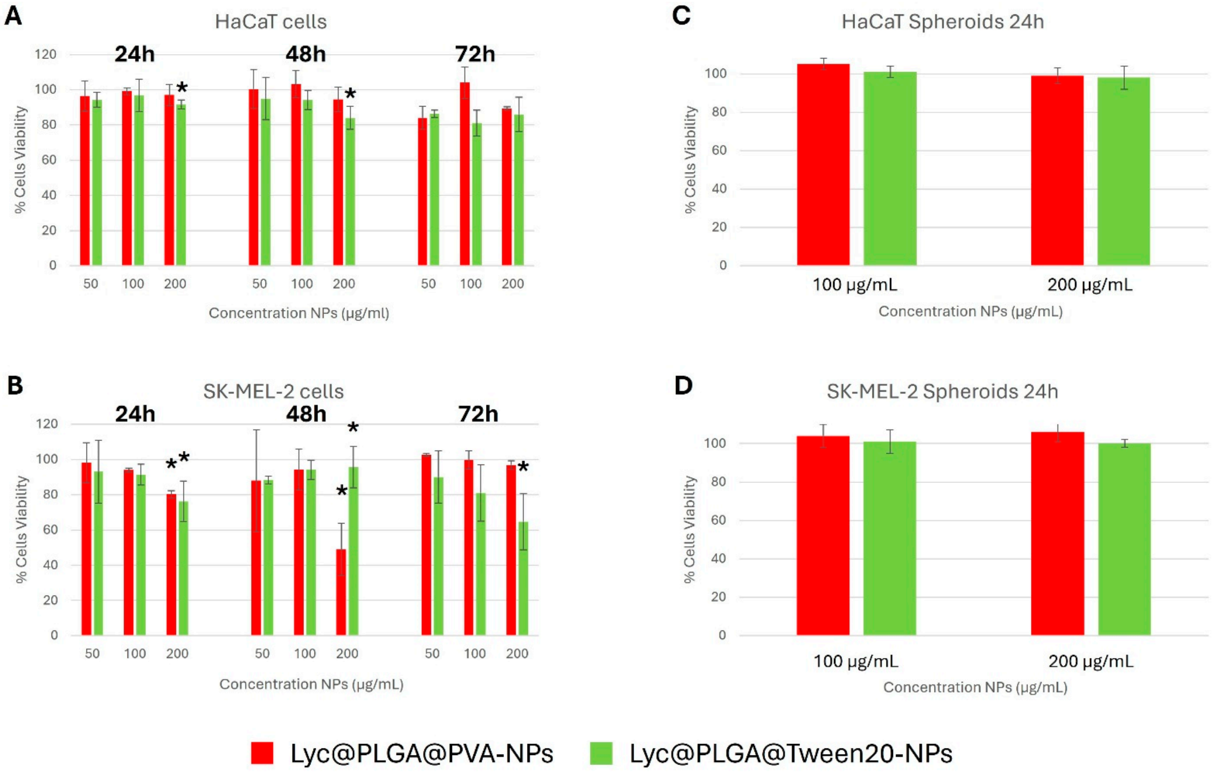

Figure S6. Cells viability percentages of HaCaT (A) and SK-MEL-2 (B) 2D culture cells after Lyc@PLGA@PVA-NPs and Lyc@PLGA@Tween 20-NPs treatment at different concentrations over time (24-48-72h); Cells viability percentages of HaCaT (C) and SK-MEL-2 (D) spheroids after Lyc@PLGA@PVA-NPs and Lyc@PLGA@Tween 20-NPs treatment at different concentrations (100-200 µg/mL) for 24h. Statistically significant value (∗) P ⩽ 0.05 *versus* not treated condition, from the t-test.

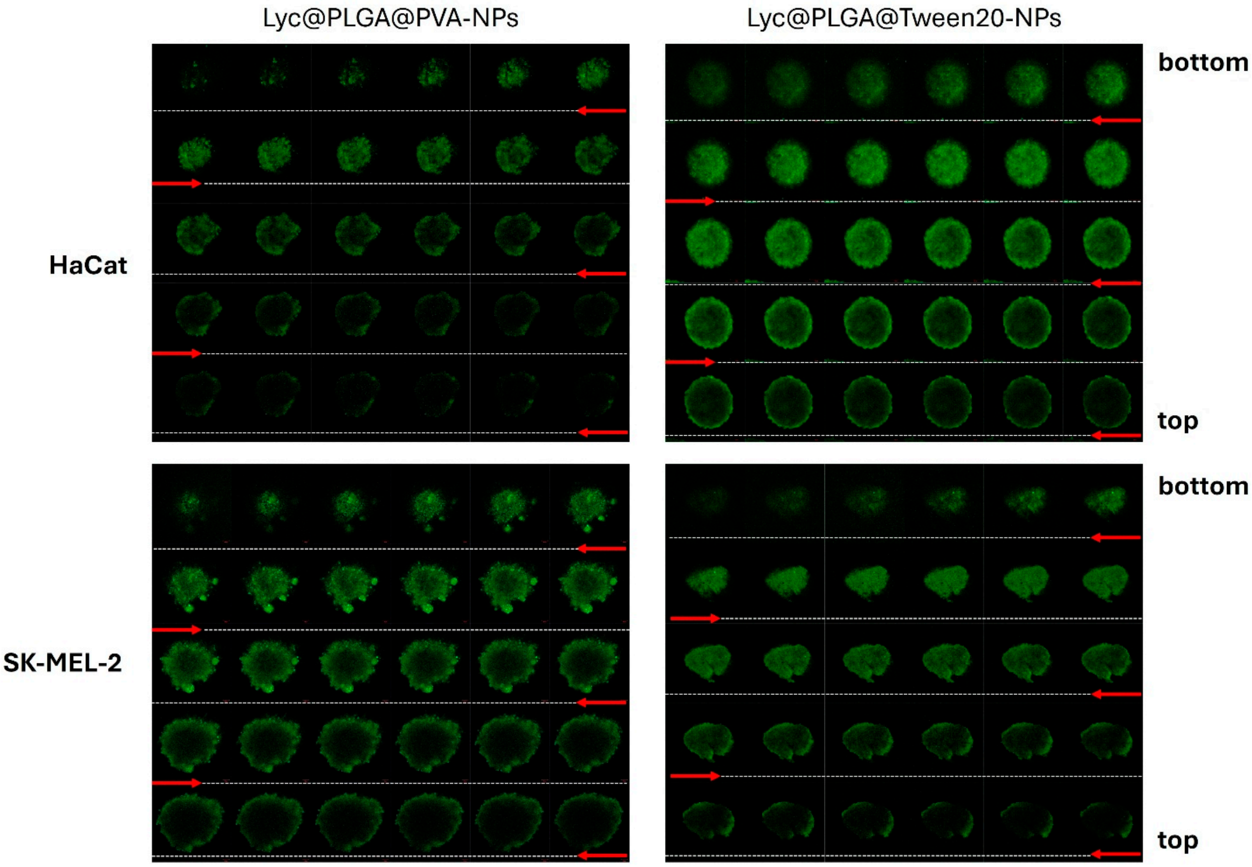

Figure S7. Representative gallery images by confocal microscopy analysis of HaCaT and SK-MEL-2 spheroids following 24 h of treatment with Lyc@PLGA@PVA-NPs and Lyc@PLGA@Tween20-NPs. 30 z stacks have been acquired for each spheroid. The figure shows the z stacks from the top and the bottom of the spheroid, with red arrows and dotted lines which indicate their order; in green emitted fluorescence of Lyc ($\lambda_{exc.}$ = 488 nm).

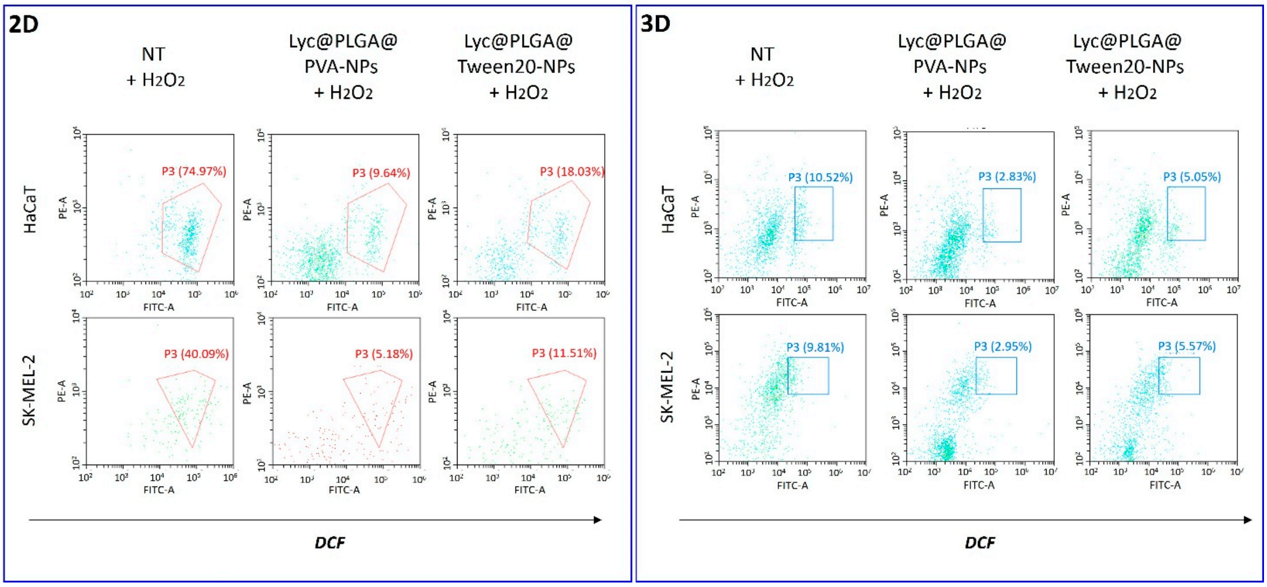

Figure S8. Representative flow cytometric spectra of intracellular ROS analysis in HaCaT and SK-MEL-2 cells and spheroids following exposure to H_2O_2 and treatment with Lyc@PLGA nanoparticles formulated with either PVA or Tween-20. Dot plots display FITC-A fluorescence on the x-axis, corresponding to DCF generated from the oxidation of the DCFH-DA probe, indicating intracellular ROS levels. The y-axis represents PE-A fluorescence (phycoerythrin), used to identify the gated cell population. The polygonal gates (P3) delineate the proportion of cells positive for ROS within the selected population. Comparisons across treatments (NT + H_2O_2, Lyc@PLGA@PVA-NPs + H_2O_2, and Lyc@PLGA@Tween20-NPs + H_2O_2) illustrate the relative shifts in DCF fluorescence, reflecting differences in oxidative stress induced or mitigated by nanoparticle formulations.
